# Previous pregnancy loss and gestational cardiovascular health: A prospective cohort of nulliparous women

**DOI:** 10.3389/fpubh.2023.1071706

**Published:** 2023-04-11

**Authors:** Shuang-shuang Ma, Wan-jun Yin, Peng Wang, Hai-xia Wang, Lei Zhang, Xiao-min Jiang, Ying Zhang, Ruixue Tao, Jin-fang Ge, Peng Zhu

**Affiliations:** ^1^Department of Sleep Disorders, Affiliated Psychological Hospital of Anhui Medical University, Hefei, China; ^2^Hefei Fourth People's Hospital, Hefei, China; ^3^Anhui Mental Health Center, Hefei, Anhui, China; ^4^Department of Maternal, Child and Adolescent Health, School of Public Health, Anhui Medical University, Hefei, Anhui, China; ^5^MOE Key Laboratory of Population Health Across Life Cycle, Anhui Medical University, Hefei, Anhui, China; ^6^NHC Key Laboratory of Study on Abnormal Gametes and Reproductive Tract, Anhui Medical University, Hefei, Anhui, China; ^7^Anhui Provincial Key Laboratory of Population Health and Aristogenics, Anhui Medical University, Hefei, Anhui, China; ^8^Department of Obstetrics and Gynecology, Anhui Women and Child Health Care Hospital, Hefei, Anhui, China; ^9^Department of Obstetrics and Gynecology, First Affiliated Hospital of Anhui Medical University, Hefei, Anhui, China; ^10^Department of Obstetrics and Gynecology, Hefei First People's Hospital, Hefei, Anhui, China; ^11^School of Pharmacy, Anhui Medical University, Hefei, Anhui, China

**Keywords:** pregnancy loss, cardiovascular health, gestation, inflammation, nulliparous women

## Abstract

**Objectives:**

To estimate the association of previous pregnancy loss with subsequent cardiovascular health during gestation and to examine the role of high-sensitivity C reactive protein (hs-CRP) in the association.

**Methods:**

A total of 2,778 nulliparous pregnant women were recruited between March 2015 and November 2020 in Hefei city, China. Their cardiovascular health (CVH) including prepregnancy body mass index (BMI), blood pressure, total cholesterol, fasting plasma glucose, and smoke status were recorded at 24–28 weeks’ gestation, as well as their reproductive history. Multivariate linear and logistic regression were performed to examine the association of pregnancy loss with cardiovascular health. And the role of hs-CRP between pregnancy loss and CVH was assessed by the mediation analysis.

**Results:**

Compared with women who have no pregnancy loss, women with a history of spontaneous or induced abortions had higher BMI (*β*, 0.72, 95% *CI*, 0.50 to 0.94) and fasting plasma glucose (*β*, 0.04, 95% *CI*, 0.01 to 0.07), and had lower total CVH scores after adjusting for confounders (*β*, −0.09, 95% *CI*, −0.18 to −0.01). CVH scores were most significantly decreased among women with 3 or more induced abortions (*β*, −0.26, 95% *CI*, −0.49, −0.02). The contribution of pregnancy loss to poorer gestational CVH mediated by increased hs-CRP levels was 23.17%.

**Conclusion:**

Previous pregnancy loss was associated with poorer cardiovascular health during gestation, which may be mediated by their gestational inflammatory status. Exposure to miscarriage alone was not a significant predictor of poorer CVH.

## Introduction

Cardiovascular disease (CVD) is the leading cause of mortality in both men and women and accounts for one-third of all deaths worldwide (1). In 2010, the American Heart Association proposed a project about cardiovascular health (CVH) scores, which was expected to reduce the CVD burden by 20% and increase cardiovascular health by 20% in 2020 (2). Currently, CVH has been a global, positive health-promotion construct that has proven widely applicable across clinical settings. Data from the Framingham Offspring Study demonstrates that the decreased ideal CVH scores over the past 20 years result in increasing odds of subclinical disease and risk of CVD death, emphasizing the importance of maintaining ideal CVH across the life course (3, 4).

Pregnancy poses a significant physiological challenge to the cardiovascular system of the mothers (5), which is a critical window to identify women with metabolic syndromes and elevated risk of adverse pregnancy outcomes, as well as later life cardiovascular diseases (6). Evidence from the Hyperglycemia and Adverse Pregnancy Outcome (HAPO) Study and HAPO Follow-Up Study suggested that better maternal CVH in gestation was associated with lower risk of adverse pregnancy outcomes (7) and better offspring CVH in early adolescence (8). Identifying risk factors for cardiovascular health or subclinical disease to highlight priorities for intervention is needed urgently. Growing evidence indicates that reproductive factors unique to women are associated with long-term risk of CVD morbidity and mortality (9–11).

Miscarriage is one of the most common complications, with an estimated prevalence of 20% in clinically recognized pregnancies ending in miscarriage (12, 13), 35 induced abortions occurred annually per 1,000 women aged 15–44 years worldwide in 2010–2014 (14). A history of pregnancy loss (PL), whether induced or naturally occurring, has been associated with a higher risk of subsequent metabolic disorders, including cardiovascular disease (15, 16), premature mortality (17), type 2 diabetes (18, 19) and atherosclerotic disease (20), especially when there is recurrent PL. Moreover, a prospective longitudinal study revealed an important temporal relationship that a delayed and more prolonged increased risk of CVD is associated with a first pregnancy loss (21). However, to the best of our knowledge, the early-term effects of PL on gestational cardiovascular health remain uncertain. It is plausible to interpret the association by biological pathways that abnormal neuroendocrine responses, proinflammatory state and unhealthy behaviors triggered by highly stressful life events (22). High sensitivity C-reactive protein (hs-CRP) is an acute phase reactant protein released from the liver and serves as a marker to identify inflammation, often used as a component of routine cardiovascular disease risk assessment (23, 24), but the role of hs-CRP in the association between a history of past PL and gestational cardiovascular health in pregnant women still needs to be investigated.

Therefore, we evaluated the association of pregnancy loss, including miscarriage and induced abortion, with gestational CVH in nulliparous pregnant women enrolled in a prospective cohort study. We further examined the extent to which this relation is mediated by hs-CRP.

## Methods

### Study design and population

The Maternal and Infant Health cohort study in Hefei (MIH-Hefei) is an ongoing prospective cohort established in March 2015, which was designed to evaluate the impact of prenatal complex setting of nutrition, stress or behaviors on adverse maternal and fetal pregnancy outcomes. Pregnant women with singleton pregnancy aged ≥18 years who had lived in Hefei for at least 2 years were were enrolled at 16 to 23 weeks’ gestation at three prenatal checkup hospitals in Hefei, China (Hefei First People’s Hospital, Anhui Maternal and Child Care Hospital, and the First Affiliated Hospital of Anhui Medical University). A face-to-face interview was used to obtain the demographic data, health status and lifestyle and dietary habits. All eligible pregnant women were examined at a target of 26 weeks’ gestation (range, 24–28) and 34 weeks’ gestation (range, 32–36), respectively. Newborns were examined within 72 h of delivery, and data were collected through pregnancy and 6–36 weeks postpartum.

As of November 2020, a total of 9,081 pregnant women were enrolled. In the current study, our analytical sample was restricted to those pregnant women who were nulliparous women at recruitment (*n* = 6,114). Women were excluded if they had liver, renal, or thyroid dysfunction (*n* = 56); prepregnancy diabetes, hypertension or cardiovascular disease (*n* = 43). Considering the risk of women’s age for miscarriage (25), women aged 35 and older were excluded (*n* = 189). And their blood samples were collected at 24–28 week’s gestation for examination. Through laboratory examination, participants without CVH data (*n* = 64) and who did not obtained blood samples (*n* = 32) were excluded. Therefore, we obtained full data, including blood samples from 5,730 nulliparous women. We randomly selected half of the blood samples (2,865 samples) were assayed for maternal hs-CRP levles, in which 87 samples were excluded (lower limit of detection). Finally, 2,778 eligible participants were analyzed (Supplementary Figure S1). All participants provided written informed consent. The study was conducted in full accordance with the Helsinki Declaration. Ethical approval was granted by the ethics committee of Anhui Medical University (No. 2015002).

### Data on pregnancy loss history

At 16–23 weeks’ gestation, their lifetime history of pregnancies (included the number of pregnancy losses, miscarriage and induced abortions) were collected in face-to-face by obstetricians at clinics. Their reproductive histories prior to this pregnancy were determined by maternal reports and verified by medical records. In our study, miscarriage is defined as a spontaneous loss of an intrauterine pregnancy prior to 24 weeks of gestation (26). Recurrent miscarriage is defined as at least three consecutive miscarriages before 24 weeks gestation (27). Induced abortion is a simple health care intervention that can be effectively managed by a broad range of health workers using medication or a surgical procedures (28).

### Assessment of cardiovascular health during pregnancy

As in prior studies (7, 8), CVH was characterized using the combination of the following 5 metrics: body mass index (BMI), blood pressure (BP), total cholesterol (TC), glucose, and smoking status. Each CVH metric was evaluated at 24–28 weeks’ gestation and was coded as a 3-level variable: ideal (2), intermediate (1), or poor (0) as described in the Supplementary Table S1. Height, weight and blood pressure were each measured twice by trained study personnel using calibrated instruments. Gestational BMI was calculated as weight (kg) divided by height (m) squared, and classified as ideal (≤28.4 kg/m^2^), intermediate (28.5–32.9 kg/m^2^), or poor (≥33.0 kg/m^2^) (29). Their blood pressure was also classified according to the pregnancy guideline (30). A 75-g oral glucose tolerance test (OGTT) was performed in the 12 h fasting state for assessment of fasting, 1- and 2-h plasma glucose levels, and poor glucose was defined as women with gestational diabetes mellitus utilizing International Association of Diabetes in Pregnancy Study Groups criteria (31). For TC concentration, we defined total cholesterol concentrations less than 260 mg/d as ideal, 260 to 299 mg/d as intermediate, and 300 mg/d or greater as poor (32). Smoking was reported from standardized questionnaires, but there was no smoking during pregnancy in this study. Thus, we used 4 metrics of the CVH to calculate a CVH score (ranged from 0 to 8), the higher CVH score, the better cardiovascular health status. And the total CVH score was categorized into 4 mutually exclusive groups: all ideal metrics, 1 or more intermediate (but 0 poor) metrics, 1 poor metric, or 2 or more poor metrics.

### Examination of hs-CRP levels

Hs-CRP has been widely used in studies to evaluate low-grade systemic inflammation in individuals (33). A venous blood sample was taken at 24–28 weeks’ gestation to test for hs-CRP levels. The level of hs-CRP was measured using enzyme-linked immunosorbent assay (ELISA) kits (Cusabio Biotech, Wuhan, China). The intra- and inter-coefficients of variation were < 10%.

### Covariates assessment

The following potential confounders were obtained from interviews included maternal demographics (age, education, family income), paternal smoking and alcohol consumption, and maternal folic acid supplements intakes. In addition, their reproductive related characteristics, pregnancy complications, gynecological-related disease, parental history of chronic diseases and their pregnancy intention were collected from interviews and medical records. Gestational age was estimated based on the last menstrual period and ultrasound assessment at baseline. Prepregnancy body mass index (BMI) was calculated by prepregnancy weight (kg)/height (m)^2^. Frequencies of moderate physical activity in the past 3 months were evaluated by the International Physical Activity Questionnaire (<3 and ≥ 3 days/week of no<10 min per day) (34). Food consumption frequency and serving size in the past month were collected using the food frequency questionnaire (FFQ). Based on 39 pre-defined food groups in our study, the relevant food groups were selected and divided into six beneficial food groups (included vegetables, legumes, fruits and nuts, cereal, fish, and dairy) and one detrimental food group (meat). A Mediterranean diet score was based on that devised by Chatzi et al. for pregnant women (35). When pregnant women whose consumption of the beneficial food groups was above the median scored 1 point, and those below the median scored 0 point. The opposite is true for meat consumption. The MD score was calculated by summing the values of the seven food groups which ranged from 0 to 7, with a higher score representing greater adherence to a Mediterranean-style diet. The details of Mediterranean diet score assessment have been described in our recent study (36). Sleep duration was recorded as the number of hours of sleep reported by asking, ‘How many hours of sleep did you generally get at night during the previous month?’. Their mental conditions were assessed using the Edinburgh Postnatal Depression Scale (EPDS) (Chinese version), a 10-item self-reporting questionnaire with well validated (37). Depression symptoms were defined as EPDS scores greater than 12.

### Statistical analysis

Data on characteristics of the participants were presented as means (standard deviation [SD]) or median (interquartile range, IQR) for continuous variables and proportions for categorical variables. Statistical analysis was perform using Student’s t-test or Mann–Whitney U-test for continuous data and chi-squared test for categorical data. Participants were considered to have been exposed to pregnancy loss from the first report of a spontaneous or induced abortion, and participants with the first gravid were considered as the reference group. Multivariable linear or logistic regression models were constructed to estimate *β* or relative risk (*RR*) and 95% confidence intervals (*CI*) to explore the associations between pregnancy loss, overall and according to the number of miscarriages and induced abortions, and CVH status after adjustment for covariates. We then tested for the role of hs-CRP levels in the association of pregnancy loss with CVH status through a mediation analysis using the SPSS PROCESS plug-in. The Bootstrap 95% CI dose not include 0 as an estimated significant indirect effect. A sensitivity analysis was performed to compare differences in characteristics between the included and excluded samples. All analyses were conducted by using Statistical Product and Service Solutions, version 23.0 (IBM Corporation, Chicago, IL). A 2-sided *p*-value of 0.05 was deemed statistically significant.

## Results

### Participants characteristics

Sample characteristics are presented in Table 1. A total of 2,778 nulliparous women were included in the study, 43.8% had at least one pregnancy ending in pregnancy loss, and of which 199 (16.4%) reported a miscarriage, and 1,017 (83.6%) induced abortion. Women who experienced pregnancy loss were older than women who never had a pregnancy loss (mean [SD], 26.86 [2.53] vs. 28.54 [3.12] years), had higher prepregnancy BMI (20.77 [2.58] vs. 21.50 [3.08] kg/m^2^) at baseline. And they were more likely to be infected with genital tract infection (12.5% vs. 9.5%) and to use progesterone (36.3% vs. 28.6%). In addition, they had higher prevalence of a parental history of diabetes (10.1% vs. 6.7%) and heart disease (4.9% vs. 2.8%), and might need longer time for pregnancy (15.0% vs. 11.3%). Of note, participants excluded due to missing data were similar to those included in our analysis in terms of socio-demographic characteristics (Supplementary Table S2).

**Table 1:** Participants characteristics according to pregnancy loss (*N* = 2,778).

Characteristics	No pregnancy loss (*n* = 1,562)	Pregnancy loss (*n* = 1,216)	*p* value
*Demographics*			
Age, years, mean (SD)	26.86 (2.53)	28.54 (3.12)	<0.001
Education level (≥12 years), No(%)	1,119 (71.6)	698 (57.4)	<0.001
Household income (≥6,000 RMB/m), No(%)	1,057 (67.7)	867 (71.3)	0.040
*Pregnancy lifestyle factors* ^a^			
Physical activity (≥3 d/w), No(%)	718 (46.0)	553 (45.5)	0.797
MeDiet score ^b^ (≥4), No(%)	1,150 (75.3)	850 (71.8)	0.042
Sleep duration (≥9 h/d), No(%)	685 (43.9)	530 (43.6)	0.887
Depressive symptom ^c^ (≥13), No(%)	137 (8.8)	104 (8.6)	0.893
Paternal alcohol intakes (≥3 times/w), No(%)	126 (8.1)	178 (14.6)	<0.001
Paternal smoking behaviors (≥6 cigarettes/d), No(%)	209 (13.4)	297 (24.4)	<0.001
Pre-pregnancy folic acid supplement (≥3 times/w), No(%)	724 (46.4)	523 (43.0)	0.079
Early-pregnancy folic acid supplement (≥3 times/w), No(%)	1,354 (86.7)	1,030 (84.7)	0.138
*Maternal Health status*			
Pre-pregnancy BMI ^d^, kg/m^2^, mean (SD)	20.77 (2.58)	21.50 (3.08)	<0.001
Family history of hypertension, No (%)	485 (31.0)	416 (34.2)	0.077
Family history of diabetes, No (%)	105 (6.7)	123 (10.1)	0.001
Family history of heart disease, No (%)	44 (2.8)	60 (4.9)	0.004
Genital tract infection, No(%)	149 (9.5)	152 (12.5)	0.013
Pelvic inflammatory disease, No(%)	38 (2.4)	48 (3.8)	0.039
*Reproductive characteristics*			
Gestational age, weeks, mean (SD)	24.40 (2.20)	24.52 (2.16)	0.175
Unintended pregnancy, No (%)	555 (35.5)	461 (37.9)	0.196
Time to pregnancy (>6 months), No (%)	177 (11.3)	182 (15.0)	0.005
Progesterone treatment, No (%)	459 (29.4)	456 (37.5)	<0.001

MeDiet, Mediterranean Diet; BMI, body mass index. Continuous data are presented as mean (standard deviation) or median (interquartile range, IQR), and categorical data as number (percentage). All variables (except fetal sex and birth outcomes) were collected at 24–28 weeks’ gestation. *p* value is from analysis of variance (for means) or chi-square (for proportions) comparing study arms.

a Pregnancy lifestyle refers to lifestyle during the 3 months before enrollment.

b The MeDiet score was evaluated by a food frequency questionnaire, including vegetables, fruits, grains, nuts, beans, fish, oil, red or processed meat and wine.

c Depression status was evaluated using the Edinburgh Postnatal Depression Scale.

d BMI was calculated as weight in kilograms divided by height in meters squared.

### Associations of pregnancy loss with CVH metrics and hs-CRP levels

Table 2 shows an association of pregnancy loss with CVH *metrics* and hs-CRP levels. The mean total gestational CVH score was 6.96 [1.2] out of 8, and 24.8% participants had ≥1 poor CVH metric in the 1,216 pregnant women with PL. Multivariate analyses demonstrated a significant decrease in maternal CVH scores (*β*, −0.09, 95% *CI*, −0.18 to −0.01) and increase in hs-CRP levels (*β*, 0.05, 95% *CI*, 0.02 to 0.08) in pregnant women who experienced PL compared with those who were no PL. For each of CVH metric, pregnant women who experienced PL had significantly higher BMI (*β*, 0.72, 95% *CI*, 0.50 to 0.94), fasting glucose (*β*, 0.04, 95% *CI*, 0.01 to 0.07), 1-h OGTT glucose (*β*, 0.27, 95% *CI*, 0.15 to 0.40) and 2-h OGTT glucose concentration (*β*, 0.21, 95% *CI*, 0.12 to 0.31).

**Table 2:** Adjusted associations of pregnancy loss with gestational cardiovascular health metrics at 24–28 weeks’ gestation.

CVH metrics	No pregnancy loss	Pregnancy loss	Adjusted model^a^	
	(*n* = 1,562)	(*n* = 1,216)	*β*(95% CI)	
*BMI*, kg/m^2^, mean (SD)	23.68 (2.76)	24.46 (3.05)	0.72 (0.50,0.94)	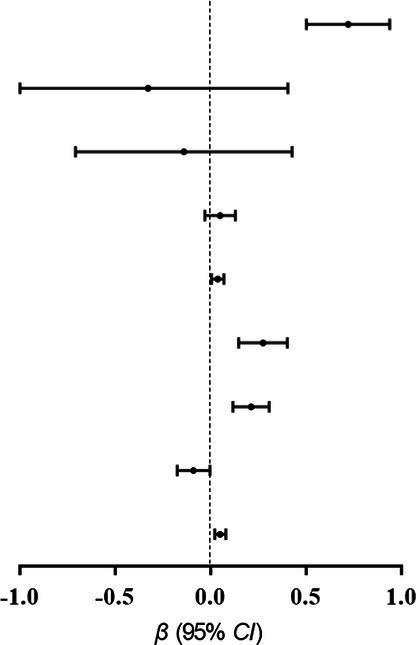
*SBP*, mm Hg, mean (SD)	110.31 (9.88)	109.94 (9.38)	−0.33(−1.06,0.41)	
*DBP*, mm Hg, mean (SD)	69.05 (7.57)	68.87 (7.35)	−0.14(−0.71,0.43)	
*TC*, mmol/L, mean (SD)	5.99 (1.04)	6.04 (1.07)	0.05(−0.03,0.13)	
*FPG*, mmol/L, mean (SD)	4.53 (0.43)	4.57 (0.45)	0.04 (0.01, 0.07)	
*1-h OGTT*, mmol/L, mean (SD)	7.14 (1.65)	7.45 (1.72)	0.27 (0.15,0.40)	
*2-h OGTT*, mmol/L, mean (SD)	6.25 (1.23)	6.47 (1.27)	0.21 (0.12, 0.31)	
*CVH score* ^b^, mean (SD)	7.09 (1.11)	6.96 (1.21)	−0.09(−0.18,-0.01)	
*Hs-CRP* ^c^, Median (IQR)	0.35 (0.08–0.60)	0.42 (0.15–0.67)	0.05 (0.02,0.08)	


CVH, cardiovascular health; BMI, body mass index; SBP, systolic blood pressure; DBP, diastolic blood pressure; TC, total cholesterol; FPG, fasting plasma glucose, OGTT, oral glucose tolerance test; Hs-CRP, high-sensitivity C reactive protein. Continuous data are presented as mean (standard deviation) or median (interquartile range, IQR).

a All estimates were adjusted for maternal age, education, household income, paternal smoking status, pre-pregnancy body mass index, time-to-pregnancy, gestation age at enrollment, physical activity, Mediterranean diet score, sleep duration, depressive symptoms, genital tract infection, pelvic inflammatory disease, progesterone treatment, family history of diabetes, hypertension and heart disease.

b The CVH score has a possible range of 0 to 10 points for pregnant mothers (observed range, 2–8 points).

c Hs-CRP index was log10-transformed to improve normality.

### Association of pregnancy loss categories with CVH status and hs-CRP levels

We jointly classified participants according to their history of miscarriage and induced abortion to test the relations of pregnancy loss with CVH status and hs-CRP levels (Table 3). Compared with women who had no PL, women with 3 or more pregnancy losses had an 1.58 (95% *CI*, 1.04 to 2.42) fold higher poor CVH status and had a significant association with increasing hs-CRP levels. And women with 3 or more induced abortions had an almost 1.67 (95% *CI*, 1.06 to 2.62) fold higher poor CVH status than the group who were no PL, with a significant decrease in CVH scores (*β*, –0.26, 95% *CI*, −0.49 to −0.02). Additionally, there was a significant increase in maternal hs-CRP level in the group with 3 or more induced abortions (*β*, 0.10, 95% *CI*, 0.02 to 0.18). We did not observe a role for hs-CRP levels in the association between miscarriage and CVH scores. As shown as Supplementary Table 3, further analyses revealed a significant associations gestational BMI and blood glucose in women with pregnancy loss or induced abortions. And as shown in Supplementary Table 4, compared to women who had no history of pregnancy loss, poorer CVH status and CVH scores were significantly different for women with a history of abortion only and one or more PL.

**Table 3:** Adjusted associations of pregnancy loss with gestational cardiovascular health status and hs-CRP levels, according to the number and type of pregnancy loss^a^.

History of pregnancy losses	CVH status^b^						hs-CRP levels^c^		
	≥1 Poor CVH metric			CVH scores					
	*n*(%)	*RR* (95% *CI*)	*p-*value ^d^	Mean (SD)	*β* (95% CI)	*p*-value	Median (IQR)	*β* (95% CI)	*p*-value
*Never pregnancy loss* (*n* = 1,562)	306 (19.6)	1.00 (Ref.)		7.10 (1.11)	Ref.		0.35 (0.08, 0.60)	Ref.	
*Miscarriage (1–2)* (*n* = 185)	44 (23.8)	1.20 (0.83, 1.72)	0.333	6.99 (1.14)	−0.07(−0.24, 0.11)	0.435	0.41 (0.18, 0.72)	0.06 (0.00, 0.12)	0.048
*Miscarriage (≥3)* (*n* = 14)	4 (28.6)	1.53 (0.473, 4.98)	0.480	7.00 (1.30)	−0.05(−0.65, 0.55)	0.874	0.49 (0.16, 0.74)	0.03(−0.18, 0.23)	0.298
*Induced abortion (1–2)* (*n* = 918)	222 (24.2)	1.24 (1.02, 1.51)	0.035	6.99 (1.20)	−0.08(−0.17, 0.02)	0.105	0.40 (0.15, 0.67)	0.05 (0.01, 0.08)	0.005
*Induced abortion (≥3)* (*n* = 99)	31 (31.3)	1.67 (1.06, 2.62)	0.028	6.79 (1.25)	−0.26(−0.49, −0.02)	0.031	0.47 (0.15, 0.70)	0.10 (0.02, 0.18)	0.021
*Pregnancy loss (1–2)* (*n* = 1,103)	266 (24.1)	1.19 (0.98, 1.44)	0.073	7.00 (1.21)	−0.04(−0.19, 0.02)	0.114	0.41 (0.15, 0.66)	0.06 (0.02, 0.08)	0.023
*Pregnancy loss (≥3)* (*n* = 113)	35 (31.0)	1.58 (1.04, 2.42)	0.036	6.73 (1.30)	−0.06(−0.65, −0.11)	0.047	0.48 (0.15, 0.70)	0.09 (0.02, 0.17)	0.048

CVH, cardiovascular health; Hs-CRP, high-sensitivity C reactive protein. Data are *n* (%) or mean (SD), unless otherwise specified.

a All estimates were adjusted for maternal age, education, household income, paternal smoking status, pre-pregnancy body mass index, time-to-pregnancy, gestation age at enrollment, physical activity, Mediterranean diet score, sleep duration, depressive symptoms, genital tract infection, pelvic inflammatory disease, progesterone treatment, family history of diabetes, hypertension and heart disease.

b CVH score has a possible range of 0 to 10 points for pregnant mothers (observed range, 2–8 points). Total CVH was also categorized into 4 mutually exclusive groups: all ideal metrics, 1 or more intermediate (but 0 poor) metrics, 1 poor metric, or 2 or more poor metrics.

c Hs-CRP index was log10-transformed to improve normality.

d *p*-value is evaluated for the relative risk of poor CVH (including 1 poor CVH metric and 2 or more poor CVH metrics) among study arms.

### Mediation analyses: Indirect effect of hs-CRP levels on CVH status

**Figure 1:**
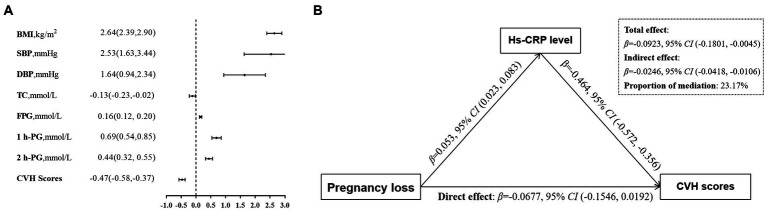
Associations of maternal blood markers with gestational CVH. **(A)** βwith 95% *CI* for the continuous outcomes of the log10-transformed maternal serum hs-CRP levels, are for CVH status including gestational BMI, SBP and DBP, TC, FPG, 1 h-PG, 2 h-PG, and CVH scores. **(B)** Mediation effects of maternal hs-CRP (log10 transformed) on the relationship between previous pregnancy loss and the CVH scores. Panels **(A,B)** adjusted confounders including maternal age, education, household income, paternal smoking status, prepregnancy BMI, time-to-pregnancy, gestation age at enrollment, physical activity, Mediterranean diet score, sleep duration, depressive symptoms, genital tract infection, pelvic inflammatory disease, progesterone treatment, family history of diabetes, hypertension and heart disease. CVH, cardiovascular health; hs-CRP, high-sensitivity C reactive protein; BMI, body mass index; SBP, systolic blood pressure; DBP, diastolic blood pressure; TC, total cholesterol; FPG, fasting plasma glucose; 1 h-PG, 1 h plasma glucose; 2 h-PG, 2 h plasma glucose.

As shown in Figure 1A, there is a negative relationship between maternal serum hs-CRP levels and CVH scores. In multivariable analysis, maternal hs-CRP was significantly associated with maternal CVH scores (*β*, −0.47, 95% *CI*, −0.58 to −0.37), as well as each of CVH metrics. Further mediation analysis demonstrated that the contribution of maternal PL to decreased CVH scores mediated by the log10-transformed maternal serum hs-CRP (Indirect effect, −0.0246 95% *CI*, –0.0418 to –0.0106), the proportions of indirect effects was 23.17% (Figure 1B).

## Discussion

We investigated the association between pregnancy loss and maternal CVH status in a prospective cohort study. Among those with a history of pregnancy loss, the CVH status was poorer than among those without pregnancy loss, and these associations were mediated by increased hs-CRP levels, with an indirect effect ratio of 23.2 percent. The risk of poorer CVH increased with the number of pregnancy losses, but a significant association was found only in participants who had only induced abortion. These findings further highlight how adverse reproductive events as early chronic stressors can lead to a higher risk of poor cardiovascular health.

Our findings are consistent with the previous findings suggesting that a history of pregnancy loss is associated with a greater risk of poor cardiovascular health. Current evidence mainly comes from retrospective cohorts and disease registry databases (38–40), most of which have reported a higher CVD risk in women with pregnancy loss. Similarly, prospective cohort studies also revealed that women with reported exposure to pregnancy loss had a greater risk of CVD than women without pregnancies ending in pregnancy loss (38, 41). Conversely, in a Japanese cohort of 54,652 women aged 40–79, women with recurrent pregnancy loss only had a slightly lower risk of death from total stroke, intracerebral haemorrhage, and total cardiovascular disease, but the risk of death from ischemic stroke increased two-fold among younger women aged 40–59 (42). The disputed results are likely due to the fact that these previous studies either determined pregnancy history at a single time point in the distant past through a hospital register database or a retrospective recall, which could have led to exposure misclassification.

We also noticed an increasing trend of higher risk for poorer CVH status with greater number of pregnancy losses. The data from a nationwide China Kadoorie Biobank recruited 302,669 Chinese women aged 30–79 years showed a J-shaped relationship between the number of pregnancies and a risk of CVD (38). Additionally, the Danish register based study also reported a growing trend of higher mortality rates with an increasing number of miscarriages (43). Recurrent miscarriage is a major stressful event in a woman’s life and has been associated with adverse mental health problems, such as depression and anxiety after loss (44). Furthermore, abortion rates among women are higher in China and may be influenced by multiple factors, including social, cultural and economic conditions. To our knowledge, women with persistent excessive bleeding, haemodynamic instability, evidence of infected retained tissue, and suspected gestational trophoblastic disease were commonly recommended to offer surgical management (45). Notably, women with surgical treatment were at higher risk of complications, had severe bleeding or pain (46), and had adverse effects on their social and psychological well beings (47, 48).

The association between pregnancy loss and cardiovascular disease may be explained by underlying shared mechanistic pathways, including insulin resistance, endothelial dysfunction, or inflammatory states (49–51). Likewise, endothelial dysfunction was related to pregnancy loss by causing defects of the placenta, and was also implicated in the pathogenesis of cardiovascular, microvascular, and homeostatic dysfunction (52). Also, epidemiological evidence showed a proinflammatory state, manifested as increased levels of hs-CRP in women with a history of recurrent pregnancy loss, that has been strongly associated with future risk of cardiovascular diseases (53). In this study, we found that the association between pregnancy loss and CVH status was interpreted by an increase in hs-CRP with mediation at 23.17%. Evidence from randomized trials found that preconception-initiated daily low-dose aspirin (LDA) treatment (anti-inflammatory drugs) could increase fecundability in certain women with a recent early pregnancy loss (54). And LDA treatment may be associated with higher ratio of having a live-born male among women with 1 to 2 prior pregnancy losses, even if women were at higher levels of inflammation (55). In addition, basal hs-CRP levels may have the potential to serve as a clinical marker to identify patients at increased risk of pregnancy loss (56). Therefore, additional investigations of targeted interventions to reduce inflammation are needed to assess the effects on pregnancy outcomes.

Our findings extend the evidence from previous studies. To the best of our knowledge, this study is the first to explore whether previous pregnancy loss is associated with gestational cardiovascular health. We also reported a graded relationship between the number of induced abortions, which is similar to findings from previous studies that found a relationship between recurrent pregnancy losses and cardiovascular diseases (17, 18). Together, these findings further highlight how reproductive events throughout a woman’s lifetime can serve as early markers of increased susceptibility to poor cardiovascular health during pregnancy. Although we have found that the association of pregnancy loss with CVH may be mediated by inflammatory markers, future studies should further assess the mechanisms underlying this relationship to aid efforts to prevent poor CVH in pregnant women, such as the effects of genetic predisposition.

The strengths of our study include its prospective study design, moderately large sample size, the availability of various reproductive characteristics, and lifestyle and health-related factors that allow us to control for confounding and examine effect modification. There are also several limitations to our study. First, some pregnancy losses may have been unreported. Because miscarriage and induced abortion are often sensitive issues, the actual incidence may be under-reported, which may result in a misclassification of exposure status and underestimate the strength of the association between pregnancy loss and CVH. Second, gestational diet and physical activity data were not included in the clinical CVH definition (which are part of the AHA’s CVH definition) (57). Although CVH based on 5 metrics has precedent and may be more clinically applicable (8, 58), studies incorporating all 7 metrics are needed. Third, observational studies such as ours can only demonstrate an association, not causality. Whether pregnancy loss merely masks pre-existing risks or results in a worse CVH status is unknown. Last, the sample size of population was relative smaller, which may limit the generalizability of our findings. The replication of the study results need to be validated by other larger data sets.

In summary, an increased history and recurrence of miscarriage was associated with a higher risk of gestational cardiovascular health among Chinese pregnant women. Our findings emphasize the importance of considering a woman’s reproductive history when assessing her risk of metabolic disorders during gestation. And more frequent screening and timely intervention may help delay or prevent the onset of cardiovascular disease in women with high rates of pregnancy loss or recurrent pregnancy loss.

## Data availability statement

The original contributions presented in the study are included in the article/Supplementary material, further inquiries can be directed to the corresponding authors.

## Ethics statement

The studies involving human participants were reviewed and approved by the Ethics Committee of Anhui Medical University. The patients/participants provided their written informed consent to participate in this study.

## Author contributions

S-sM: data acquisition and analysis and writing-original draft preparation. W-jY: data acquisition and analysis and methodology. PW: data acquisition, visualization, and software. H-xW and LZ: data acquisition and investigation. X-mJ, YZ and R-xT: investigation and validation. J-fG: study conceptualization and supervision. PZ: study conceptualization, methodology, writing-reviewing and editing. All authors have reviewed the manuscript and took responsibility for the paper.

## Funding

The research received financial support from the National Natural Science Foundation of China (82173531 and 81872631), the National Key R&D Program of China (2022YFC2702901), and the Foundation for Scientific Research Improvement of Anhui Medical University (2021xkjT009).

## Conflict of interest

The authors declare that the research was conducted in the absence of any commercial or financial relationships that could be construed as a potential conflict of interest.

## Publisher’s note

All claims expressed in this article are solely those of the authors and do not necessarily represent those of their affiliated organizations, or those of the publisher, the editors and the reviewers. Any product that may be evaluated in this article, or claim that may be made by its manufacturer, is not guaranteed or endorsed by the publisher.
