# Supplementary material for: Previous pregnancy loss and gestational cardiovascular health: A prospective cohort of nulliparous women
Source: Front Public Health. 2023 Apr 11;11:1071706. doi: 10.3389/fpubh.2023.1071706 (PMC10127104; doi:10.3389/fpubh.2023.1071706)
Supplement: Supplementary file 1 [file Data_Sheet_1.docx]

| **Supplemental Table 1. Classification of Gestational Cardiovascular Health Metrics** | | | |
| --- | --- | --- | --- |
|  | **Ideal (2 points)** | **Intermediate (1 point)** | **Poor (0 point)** |
| **Body mass index**, kg/m^2^ | ≤ 28.4 | 28.5-32.9 | ≥ 33 |
| **Blood pressure**, mm Hg | SBP < 120 and DBP < 80 | SBP 120-139 or  DBP 80-89 | SBP ≥ 140 or  DBP ≥ 90 |
| **Total** **cholesterol level**, mmol/L | < 6.72 | 6.73-7.76 | ≥ 7.77 |
| **Blood glucose level**, mmol/L | All others | ⎯ | Gestational diabetes:  Fasting ≥5.1 or 1-h OGTT ≥10.0 or 2-h OGTT ≥8.5 |
| **Smoking status** | No |  | Yes |
| Abbreviations: SBP, systolic blood pressure; DBP, diastolic blood pressure; OGTT, oral glucose tolerance test. | | | |

| **Supplemental Table 2. Maternal baseline characteristics of participants included vs not included** | | | | | |
| --- | --- | --- | --- | --- | --- |
|  | |  | **Included participants** | **Excluded participants** | ***P* value** |
|  |  |  | **(n=2 778)** | **(n=3 336)** |  |
| **Demographics** | | |  |  |  |
|  | Age, years, mean (SD) | | 28.24(3.77) | 30.02(4.30) | 0.103 |
|  | Education level (≥12 years), No(%) | | 1817(65.4) | 2265(67.9) | 0.040 |
|  | Household income (≥6000 RMB/m), No(%) | | 1924(69.3) | 2367(71.0) | 0.149 |
| **Pregnancy lifestyle factors** ^a^ | | |  |  |  |
|  | Physical activity (≥3 d/w), No(%) | | 1271(45.8) | 1472 (44.1) | 0.203 |
|  | MeDiet score ^b^ (≥4), No(%) | | 2000(73.7) | 1413(76.6) | 0.028 |
|  | Sleep duration (≥9 hours/d), No(%) | | 1215(43.7) | 1525(45.7) | 0.122 |
|  | Depressive symptom ^c^ (>13), No(%) | | 241(8.1) | 301(9.0) | 0.634 |
|  | Husband’s alcohol intakes (≥3 times/w), No(%) | | 304(10.9) | 343(10.3) | 0.403 |
|  | Husband’s smoking behaviors (≥6 cigarettes/d), No(%) | | 506(18.2) | 510(15.3) | 0.002 |
|  | Prepregnancy folic acid supplement (≥3 times/w), No(%) | | 1247(45.3) | 1511(45.3) | 0.751 |
|  | Early-pregnancy folic acid supplement (≥3 times/w), No(%) | | 1880(85.1) | 3329(85.2) | 0.930 |
| **Maternal Health status** | | |  |  |  |
|  | Prepregnancy BMI ^d^, kg/m^2^, mean (SD) | | 21.16(2.82) | 21.14(2.89) | 0.840 |
|  | Family history of hypertension, No (%) | | 901 (32.4) | 1096(32.9) | 0.727 |
|  | Family history of diabetes, No (%) | | 228(8.2) | 299(9.0) | 0.295 |
|  | Family history of heart disease, No (%) | | 104(3.7) | 142(4.3) | 0.310 |
|  | Genital tract infection, No(%) | | 301(10.8) | 346(10.4) | 0.558 |
|  | Pelvic inflammatory disease, No(%) | | 84(3.0) | 115(3.4) | 0.353 |
| **Reproductive characteristics** | | |  |  |  |
|  | Gestational age, weeks, mean (SD) | | 24.46(2.14) | 23.56(3.52) | 0.001 |
|  | Unintended pregnancy, No (%) | | 1016(36.6) | 1285(38.5) | 0.118 |
|  | Time to pregnancy (>6 months), No (%) | | 359(12.9) | 503(15.1) | 0.016 |
|  | Progesterone treatment, No (%) | | 915(32.9) | 1065(31.9) | 0.399 |
| Abbreviations: MeDiet, Mediterranean Diet; BMI, body mass index.  Continuous data are presented as mean (standard deviation;) or median (interquartile range, IQR), and categorical data as number (percentage). *P* value is from analysis of variance (for means) or chi-square (for proportions) comparing study arms. All variables (except fetal sex and birth outcomes) were collected at 24-28 weeks’ gestation. *P* value is the difference between the two groups.  ^a^ Pregnancy lifestyle refers to lifestyle during the 3 months before enrollment.  ^b^ The MeDiet score was calculated by a food frequency questionnaire, including vegetables, fruits, grains, nuts, beans, fish, oil, red or processed meat and wine.  ^c^ Depression status was evaluated using the Edinburgh Postnatal Depression Scale.  ^d^ BMI was calculated as weight in kilograms divided by height in meters squared. | | | | | |

| **Supplementary Table 3 Adjusted associations of pregnancy loss with gestational cardiovascular health metrics and hs-CRP levels, according to the number and type of pregnancy loss ^a^** | | | | | | | |
| --- | --- | --- | --- | --- | --- | --- | --- |
|  | Pregnancy loss (0)  (n=1 562) | Miscarriage (1-2)  (n=185) | Miscarriage (≥3) (n=14) | Induced abortion (1-2) (n=918) | Induced abortion (≥3) (n=99) | Pregnancy loss (1-2) (n=1103) | Pregnancy loss (≥3) (n=113) |
| **BMI**, kg/m^2^, mean (SD | **Ref.** | 0.07(0.32,1.20)** | 0.01(-0.96,2.07) | 0.11(0.44,0.92)*** | 0.07(0.56,1.77)*** | 0.12(0.47,0.92)*** | 0.07(0.52,1.65)*** |
| **SBP**, mm Hg, mean (SD) | **Ref.** | 0.01(-1.32,1.63) | -0.03(-9.69,0.45) | -0.02(-1.28,0.32) | 0.03(-0.41,3.62) | -0.02(-1.14,0.38) | 0.02(-1.10,2.68) |
| **DBP**, mm Hg, mean (SD) | **Ref.** | -0.01(-1.49,0.76) | -0.02(-6.06,1.70) | -0.01(-0.81,0.42) | 0.04(0.18,3.27)* | -0.02(-0.81,0.35) | 0.03(-0.23,2.66) |
| **TC**, mmol/L, mean (SD) | **Ref.** | 0.03(-0.05,0.28) | -0.01(-0.59,0.53) | 0.02(-0.05,0.13) | 0.01(-0.18,0.26) | 0.02(-0.03,0.14) | 0.01(-0.18,0.24) |
| **FPG**, mmol/L, mean (SD) | **Ref.** | 0.07(0.32,1.20) | 0.02(-0.13,0.33) | 0.05(0.01,0.08)** | 0.02(-0.03,0.15) | 0.04(0.01,0.067)* | 0.03(-0.02,0.15) |
| **1-h OGTT**, mmol/L, mean (SD) | **Ref.** | 0.03(-0.06,0.45) | 0.01(-0.85,0.92) | 0.08(0.15,0.43)*** | 0.05(0.01,0.80)* | 0.08(0.14,0.41)*** | 0.05(0.67,0.72)* |
| **2-h OGTT**, mmol/L, mean (SD) | **Ref.** | 0.03(-0.02,0.36) | 0.04(0.04,1.36)* | 0.08(0.12,0.33)*** | 0.04(-0.01,0.51) | 0.08(0.16,0.31)*** | 0.05(0.06,0.55)* |
| Abbreviations: BMI, body mass index; SBP, systolic blood pressure; DBP, diastolic blood pressure; TC, total cholesterol; FPG, fasting plasma glucose, OGTT, oral glucose tolerance test. Continuous data are presented as mean (standard deviation) or median (interquartile range, IQR).  a All estimates were adjusted for maternal age, education, household income, husband smoking status, pre-pregnancy body mass index, time-to-pregnancy, gestation age at enrollment, physical activity, Mediterranean diet score, sleep duration, depressive symptoms, genital tract infection, pelvic inflammatory disease, progesterone treatment, family history of diabetes, hypertension and heart disease. * P value<0.05; ** P value<0.01; *** P value<0.001. | | | | | | | |

| **Supplementary Table 4 Adjusted association between pregnancy losses and CVH across different numbers or types of pregnancy loss ^a^** | | | | | | | |
| --- | --- | --- | --- | --- | --- | --- | --- |
| **Group** | **≥ 1 Poor CVH metric** | | |  | **CVH scores** | | |
|  | n(%) | *RR* (95% *CI*) ^b^ | *RR* (95% *CI*) ^c^ |  | Mean (SD) | *β* (95% *CI*) ^b^ | *β* (95% *CI*) ^c^ |
| No pregnancy loss (N=1562) | 306(19.6) | Ref. |  |  | 7.10(1.11) | Ref. |  |
| Types of pregnancy loss（N=1216） |  |  |  |  |  |  |  |
| Only miscarriage (n=147) | 31(21.1) | 1.097(0.724,1.662) | Ref. |  | 6.99(1.17) | -0.021(-0.305,-0.085) | Ref. |
| Only abortion (n=930) | 238(25.6) | 1.412(1.164,1.712)*** | 1.287(0.843,1.964) |  | 6.91(1.21) | -0.076(-0.280,-0.093)*** | -0.027(-0.289,0.136) |
| Both miscarriage and abortion (n=139) | 32(23.0) | 1.228(0.811,1.857) | 1.119(0.640,1.958) |  | 6.92(1.31) | -0.033(-0.375,-0.025) | -0.017(-0.348,0.217) |
| Numbers of pregnancy loss（N=1216） |  |  |  |  |  |  |  |
| Only one pregnancy loss (n=772) | 188(24.4) | 1.321(1.075,1.624)*** | 1.0 |  | 6.96(1.22) | -0.053(-0.236,-0.037)** | Ref. |
| Multiple pregnancy losses (n=444) | 113(25.5) | 1.401(1.094,1.795)*** | 1.060(0.810,1.388) |  | 6.85(1.22) | -0.078(-0.366,-0.123)*** | -0.043(-0.251,-0.034) |
| Abbreviations: CVH, cardiovascular health.  a All estimates were adjusted for maternal age, education, household income, husband smoking status, pre-pregnancy body mass index, time-to-pregnancy, gestation age at enrollment, physical activity, Mediterranean diet score, sleep duration, depressive symptoms, genital tract infection, pelvic inflammatory disease, progesterone treatment, family history of diabetes, hypertension and heart disease.  ^b^ Results were estimated by comparing a group with no history of pregnancy loss.  ^c^ Results were estimated by comparing a group with only miscarriage or a group with only one pregnancy loss.  ** P value<0.01; *** P value<0.001. | | | | | | | |

**Figure S1. Flow chart of participants throughout study.**


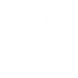


**Enrolled participants**

**6 114**

**Excluded**

**288**

**189** Age ≥ 35 years

**56** Liver, renal or thyroid dysfunction **43** History of hypertention/diabetes


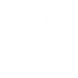


**5 826**


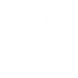


**2 865**


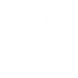


**5 730**


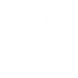


**Total included in analysis**

**2 778**


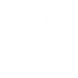


**Excluded**

**87**


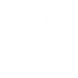


**Excluded**

**96**

**87** Below the lower detection limit (Hs-CRP)

**64** Missing CVH data

**32**  Unobtained blood samples

**Eligible participants**


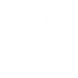


**Half of samples randomly selected**

**Eligible participants**

(16-23 gestational weeks)

(24-28 gestational weeks)
